# Administration route and trial repetition shape the effects of a commercial synbiotic on broiler production performance, cecal microbiota and pathogen colonization

**DOI:** 10.1016/j.psj.2025.106353

**Published:** 2025-12-28

**Authors:** Jana Avberšek, Aleksander Mahnič, Darja Kušar, Bojan Papić, Olga Zorman Rojs, Tomaž Knafelc, Jasna Perc, Maja Rupnik, Matjaž Ocepek

**Affiliations:** a University of Ljubljana, Veterinary Faculty, Institute of Microbiology and Parasitology, Gerbičeva 60, SI-1000 Ljubljana, Slovenia; b National Laboratory of Health, Environment and Food, Prvomajska 1, SI-2000 Maribor, Slovenia; c University of Maribor, Faculty of Medicine, Taborska 8, SI-2000 Maribor, Slovenia; d University of Ljubljana, Veterinary Faculty, Institute of Poultry, Birds, Small Mammals and Reptiles, Gerbičeva 60, SI-1000 Ljubljana, Slovenia; e VET.AM.JATA d.o.o., Slomškova 30, SI-1230 Domžale, Slovenia; f Pivka perutninarstvo d.d., Kal 1, SI-6257 Pivka, Slovenia

**Keywords:** Broiler, Synbiotic supplement, *Campylobacter jejuni*, *Salmonella* Infantis, Cecal microbiota

## Abstract

*Campylobacter* and *Salmonella* are leading causes of foodborne bacterial enteritis, with poultry meat being an important source. This longitudinal field study evaluated the impact of the commercial synbiotic PoultryStar, administered via water or feed, on broiler production performance, gut colonization with *Campylobacter jejuni* and *Salmonella* Infantis, and cecal microbiota. Two large food business operators were included in the study, with two independent trials conducted at each. A significantly higher European efficiency factor was observed when synbiotic was supplemented in water rather than in feed, although the effect did not differ from the control group. Body weight also tended to increase with synbiotic supplementation in water, although the effect was inconsistent across farms. Feed supplementation significantly reduced colonization of broilers with *S.* Infantis, whereas no effect was observed regarding *C. jejuni*. However, two bacterial taxa potentially contributing to the colonization resistance against *C. jejuni* were identified, belonging to the genus *Lactobacillus* and an unclassified representative of *Bacteroidota.* Administration of the synbiotic significantly influenced cecal microbiota, with outcomes depending on the administration route but, importantly, varying significantly between food business operators and even trial repetitions. Overall, PoultryStar exerted limited effects on pathogen control and production performance, while external factors such as administration route and trial repetition strongly modulated outcomes. Our findings highlight the need for repeated, farm-level evaluations and tailored probiotic strategies to optimize broiler health.

## Introduction

Broiler cecal microbiota is of great importance for both poultry health and food safety. This complex ecosystem plays a crucial role in the host’s digestion, metabolism and immune function, and also influences the gut colonization with pathogens such as *Campylobacter* and *Salmonella*, which are the leading cause of foodborne bacterial enteritis in humans worldwide (Kohl, 2012; EFSA and ECDC, 2023). To reduce the burden of these diseases in humans, multiple intervention approaches are being pursued in poultry meat production in response to increasing global demand for chicken meat and the growing threat of antimicrobial resistance. These efforts focus on preventing the introduction of microbial pathogens and reducing their colonization in the poultry gut. Strategies include enhanced on-farm biosecurity measures, vaccination and the use of feed and water additives such as probiotics/synbiotics, bacteriophages, bacteriocins and chemical additives (Soro et al., 2020). Measures in slaughterhouses to prevent fecal contamination of carcasses and processing strategies appear to be less effective than on-farm strategies, but they still play an important role in the holistic control of foodborne pathogens (Soro et al., 2020).

Probiotic strains most commonly used as feed supplements for chickens are certain strains of bacilli, lactobacilli, enterococci, bifidobacteria and yeasts, which can be combined with prebiotics such as galacto-oligosaccharides, fructo-oligosaccharides and xylo-oligosaccharides to form synbiotic preparations (Khan et al., 2020; Redweik et al., 2020; Lambo et al., 2021). Several studies have shown that probiotics positively influence poultry production, reduce pathogen load and promote host immunity, health and welfare. In laying hens, probiotics have been shown to improve egg quality and overall performance, whereas in broilers, they enhance growth performance and meat quality (Oh et al., 2017; Park et al., 2017; Wang et al., 2017; Abd El-Hack et al., 2020; Krysiak et al., 2021; Soumeh et al., 2021; Memon et al., 2022; Xu et al., 2023; Hoepers et al., 2024; Li et al., 2024). Such effects can be achieved either directly by modulating the gut microbiota or indirectly by modulating the host immune system (Krysiak et al., 2021).

*Salmonella* Infantis is the most common serovar in broiler farms in the EU (including Slovenia) and the fourth most common *Salmonella* serovar in human salmonellosis cases in EU/EEA countries (ECDC, 2024). Over the last decade, the *S*. Infantis lineage carrying a multidrug resistance plasmid of emerging *S*. Infantis (pESI) has replaced the pESI-free population at both national and global levels (Papić et al., 2022; Alvarez et al., 2023). Previous studies have shown a positive effect of probiotic administration on gut colonization with *Salmonella* serovars Braenderup (Zhang et al., 2022), Enteritidis (Higgins et al., 2007; Carter et al., 2017), Gallinarum (Upadhaya et al., 2016; Oh et al., 2017; Park et al., 2017), Heidelberg (Hoepers et al., 2024), Pullorum (Ma et al., 2023), Typhimurium (Higgins et al., 2007; Park and Kim, 2015) and Infantis (Šefcová et al., 2023; Drauch et al., 2025; Soffritti et al., 2025). However, it should be noted that most of these studies used different probiotic preparations and that with one exception, they were clinical trials conducted in a controlled environment with experimentally challenged animals, which may not be very representative of actual broiler production conditions.

Campylobacteriosis is the most frequently reported foodborne zoonosis in the EU (ECDC, 2024). Previous studies have shown that probiotic supplementation also reduces *Campylobacter jejuni* load in the broiler gut, but again, with one exception, these were mostly clinical trials conducted in a controlled environment (Morishita et al., 1997; Ghareeb et al., 2012; Smialek et al., 2018; Cui et al., 2024).

Recent efforts have emphasized the need for standardized workflows in poultry microbiota studies using 16S rRNA gene amplicon sequencing. Variability in sampling, storage, DNA extraction, primer selection, sequencing platforms, bioinformatic pipelines and metadata reporting limits reproducibility and cross-study comparability (Lyte et al., 2025). Standardized methodologies are particularly important when assessing microbiota-targeted interventions such as probiotics and synbiotics. In this study, we adhered to the established good practices for 16S amplicon-based microbiota research in poultry (Lyte et al., 2025) and carefully documented all experimental procedures, including study design, sampling, DNA extraction, primers, sequencing, bioinformatics and data deposition. This comprehensive documentation enables a robust assessment of synbiotic effects across farms and production cycles.

There is a lack of large-scale and comprehensive field studies that would evaluate the effects of synbiotic supplementation on broiler production parameters, pathogen load and microbiota composition. The aim of this longitudinal study was to investigate the effect of a commercial synbiotic preparation PoultryStar (Biomin, Austria) on broiler production parameters, gut colonization with *C. jejuni* and *S.* Infantis and composition of cecal microbiota. The synbiotic PoultryStar was administered to broilers via feed or water in two large food business operators (FBOs), with two independent trials being conducted in each FBO.

## Materials and methods

### Study design

Broiler flocks from two large FBOs (FBOs 1 and 2) in Slovenia were included in the longitudinal study; both FBOs reared the same chicken breed in both trials (Ross 308 Aviagen® broilers). The average flock size per barn was approx. 20,000 broilers in FBO 1 and approx. 12,000 in FBO 2. The rearing conditions and management were comparable across all barns in both FBOs and in both trials. All barns had slated floors and were equipped with automatic feeders and water system. Ventilation was provided by automatic ventilators. Prior to each placement of chickens, the houses were cleaned and disinfected, and a new litter was added. When chickens were placed in the barns, the floor temperature was > 25°C. Barn temperature was 29–32°C at the start of the production cycle and gradually decreased to 23–27°C by day 10. Broilers were reared under a standard lighting program: 23 h light and 1 h darkness during the first week, followed by 18 h light and 6 h darkness from day 8 until the day before slaughter. Chickens were fed with commercially available diets produced in the feed mill of each FBO. Three feeding mixtures were used on both FBOs: bro-starter during the first two weeks, followed by bro-finisher 1 (both with coccidiostats), and bro-finisher 2 (without coccidiostats) prior to slaughter. All diets were formulated according to the nutritional specifications and recommendations for Ross 308 broilers (Aviagen, 2019). All flocks on both FBOs were vaccinated against infectious bronchitis, Newcastle disease and infectious bursal disease according to the standard vaccination program.

The aim of the study was to assess the impact of the commercially available synbiotic PoultryStar (Biomin, Getzersdorf, Austria) on the broiler production performance, gut colonization with *C. jejuni* and *S.* Infantis and composition of cecal microbiota. According to the manufacturer’s declaration, the synbiotic contained three probiotic bacterial species (*Bifidobacterium animalis, Lactobacillus salivarius* and *Enterococcus faecium* in a ratio of 3:1:6) and a prebiotic (fructooligosaccharides). The synbiotic PoultryStar was selected by the two FBOs conducting the trials based on its feasibility for future implementation, particularly regarding cost and availability. The soluble form (PoultryStar Sol) was administered in water (W) and the microencapsulated form (PoultryStar Me) in feed (F). Protocol W included spraying of chicken at housing and supplementation in the drinking water (20 g of PoultryStar Sol per 1000 chicken) at days 2, 3, 7, 16 and 17 of the production cycle. One-day old chickens were sprayed in the barns: chickens were transferred from the boxes to the littered flood in batches and immediately sprayed with a synbiotic solution mixed with blue dye to ensure complete coverage. Protocol F included supplementation in feed (1 kg of PoultryStar Me per 1000 kg of feed) from the first to the last day of the production cycle.

For each FBO, six barns were selected, two per each study group: W, F and the control group with no supplementation. Besides the synbiotic supplementation, all other production regimens were performed in accordance with the standard FBO’s practices. Barns were selected based on their history of *C. jejuni* and *S.* Infantis colonization, meaning that broilers in these barns had been naturally colonized with *C. jejuni* and *S.* Infantis in several previous production cycles, which was confirmed with bacteriological testing according to ISO 10272-1:2017 and ISO 6579-1:2017. No antibiotics were used in any of the barns during the trials. Two separate barns were dedicated to each study group (W, F and control) to allow assessment of potential barn effects on study outcomes. In each trial, ten broilers per study group were sampled at each time point (five per barn) and analyzed individually; broilers were selected randomly across the entire barn. The following time points (corresponding to broiler age groups, AGs) were used: three days of age (AG 1), 7–8 days of age (AG 2), 19–22 days of age (AG 3) and 32–36 days of age (AG 4). Broiler cecal contents were collected on the same day. In each trial, 40 broilers originating from two barns with the same administration route were sampled per each of the three study groups, totaling 120 broilers per trial and per FBO. The trial and on-farm sampling were performed twice in each FBO, totaling 480 broiler cecal samples per study. The first trial was performed in autumn 2020 (FBOs 1 and 2) and then repeated in spring 2021 (FBO 1) or autumn 2021 (FBO 2). The trials on both FBOs were conducted as part of standard business practices during regular production cycles.

### Broiler production performance

The following broiler production parameters were monitored and calculated at slaughter in each trial for each study group: average broiler age (length of the fattening period), total animal loss, average broiler weight at the end of the production cycle, feed conversion rate (FCR) and production index evaluated as the European efficiency factor (EEF). Body weights and sex of broilers sampled at different AGs during the production cycle were also determined. Kruskal-Wallis test implemented in GraphPad Prism v8.0.2 (GraphPad Software, CA, USA) was used to statistically compare the production parameters between the study groups for each FBO. For this purpose, the values from both trials were combined for each FBO, and a *p*-value of 0.05 was considered significant.

### Sampling and DNA extraction from cecal samples

Selected broilers were euthanized by cervical dislocation and immediately dissected. The ceca were aseptically removed, with sterile gloves changed between broilers and the dissection tools and table cleaned with 70% ethanol to prevent cross-contamination. Each pair of ceca was placed in a sterile plastic container (Golias, Slovenia) and transported on ice to the laboratory within 30 min. Cecal contents were collected by cutting one cecum, and 180–220 mg of cecal contents from each broiler were transferred to a sterile Eppendorf tube, suspended in 1 ml of InhibitEX buffer and stored at –70°C until use. Total DNA was extracted using QIAamp Fast DNA Stool Mini Kit (Qiagen, Hilden, Germany) according to the adjusted manufacturer’s instructions for pathogen detection with the following modification: thawed samples were vortexed vigorously for 1 min, and 1 ml of each sample was transferred to a tube containing glass beads of ≤ 106 µm in diameter (Sigma-Aldrich, MO, USA). Samples underwent mechanical shearing at 7,000 rpm for 70 s using MagNA Lyser (Roche Diagnostics, Mannheim, Germany). Supernatant (400 µl) was transferred to sterile 1.5-ml tube, incubated at 95°C for 5 min and vortexed for 15 s. Total DNA was then extracted according to the manufacturer’s instructions, and elution of DNA was performed twice with 100 µl of ATE buffer. The extracted DNA (200 µl) was stored at –70°C until use. Sterile distilled water was used as a negative control in every batch of the samples subjected to DNA extraction. In addition, DNA was extracted from the PoultryStar Sol preparation; 1 g were supplemented with PCR-grade water to a final volume of 1 ml and the extraction was performed using the QIAamp Fast DNA Stool Mini Kit following the protocol described above.

### *Detection of Campylobacter jejuni and Salmonella* spp. *using real-time PCR*

Real-time PCR detection of *C. jejuni* (Best et al., 2003) and *Salmonella* spp. (Daum et al., 2002) was performed using real-time PCR on the Applied Biosystems 7500 Fast Real-Time PCR System (Thermo Fischer Scientific, MA, USA). For *C. jejuni*, the real-time PCR reaction mix targeting *mapA* contained 1 × Maxima Probe qPCR Master Mix and 0.3 µM ROX solution (Thermo Fisher Scientific, MA, USA), 900 nM of each primer and 250 nM of FAM-labeled probe, 3 µl of the extracted DNA and PCR-grade water to a final volume of 20 µl. Amplification of a 95-bp *mapA* fragment was performed according to the following protocol: 95°C for 10 min, and 45 cycles at 95°C for 15 s and 58°C for 1 min. For *Salmonella* spp*.*, the real-time PCR reaction mix targeting *invA* contained 1 × TaqMan Universal PCR Master Mix (Thermo Fisher Scientific, MA, USA), 500 nM of each primer and 100 nM of FAM-labeled probe, 2.5 µl of the extracted DNA and PCR-grade water to a final volume of 25 µl. Amplification of a 102-bp *invA* fragment was performed according to the following protocol: 50°C for 2 min, 95°C for 10 min, and 40 cycles at 95°C for 15 s and 60°C for 1 min.

### 16S rRNA amplicon sequencing

The extracted DNA was quantified using Quant-iT PicoGreen dsDNA Assay (Thermo Fisher Scientific, MA, USA) and normalized to 5 ng/µl. Libraries were prepared according to the 16S Metagenomic Sequencing Library Preparation guide (Illumina, CA, USA); target amplification of the V3–V4 hypervariable region of the 16S rRNA gene was performed using a broad-range set of primers S-d-Bact-0341-b-S-17 (5′–CCT ACG GGN GGC WGC AG–3′) and S-d-Bact-0785-a-A-21 (5′–GAC TAC HVG GGT ATC TAA TCC–3′) (Klindworth et al., 2013). Libraries were quality-checked using High-Sensitivity DNA kit (Agilent, CA, USA). Paired-end sequencing (2 × 300 bp) was performed on the MiSeq System (Illumina, CA, USA).

Quality filtering of reads and construction of zero-radius operational taxonomic units (ZOTUs) were performed using UNOISE3 pipeline implemented in USEARCH v.11.0.667 (Edgar, 2010; Edgar et al., 2011) with default settings. ZOTUs were taxonomically assigned using the RDP reference database v.18 (https://mothur.org/wiki/rdp_reference_files/). On average, 16,252 reads per sample were obtained (SD = 6,055). ZOTUs with an overall abundance of < 0.01% were removed, and all samples were rarefied to 8,000 reads per sample to minimize the effect of sequencing depth. Sequencing data is available on NCBI under BioProject PRJNA1014358.

Alpha and beta diversity analyses, together with the population-level analysis, were performed using mothur v.1.44.3 (Schloss et al., 2009). The remaining statistical analyses and data visualizations were performed in R v.4.0.4 using vegan (Oksanen et al., 2020) and ggplot2 (Wickham, 2016) packages. Permutational multivariate analysis of variance (PERMANOVA) was performed using the ‘adonis’ function in vegan package to test for the contribution of individual variables to the variability in bacterial community composition. Analysis of molecular variance (AMOVA) was performed in mothur to study the variation within the experimental groups W, F and control group, and to test whether genetic diversity was significantly influenced by the studied variables, namely FBO/location, trial repetition, broiler age and sex, synbiotic administration route and *C. jejuni* or *S.* Infantis colonization status. Linear discriminant analysis effect size (LEfSe) population analysis was performed in mothur to determine the differentially abundant ZOTUs by including FBO and trial as blocks.

## Results

### Effect of the synbiotic on broiler production performance

**Fig. 1:**
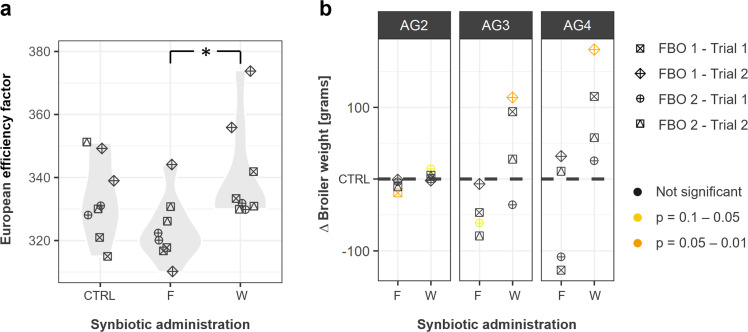
**Effect of synbiotic administration on broiler production parameters.** Parameters are presented as the European efficiency factor calculated at slaughter for the barns included in the study **(a)** and as the average broiler weight at different age groups (AGs) **(b)**. Values obtained in the study groups that were administered the synbiotic in water (W) or feed (F) were compared with the control group (CTRL) without synbiotic supplementation. Values are shown separately for each food business operator (FBO) and trial, with statistical significance color-coded as described in the legend.

The broiler production parameters, measured as cumulative values for each barn, are shown in Table 1. Sampled animals were selected randomly across the entire barn, resulting in an average male-to-female ratio of 47:53. In general, the parameters did not differ significantly between the study groups in any of the FBOs studied. A notable exception was the EEF, which was highest in the group that was administered the synbiotic in water (group W). It was significantly higher than in group F (adjusted *p* = 0.019; Kruskal-Wallis test) but not higher than in control group (adjusted *p* = 0.470; Kruskal-Wallis test) (Fig. 1**a**). In addition, a trend towards higher average weight was observed in broilers of AGs 2 and 3 from group W compared with the control group, but this increase varied between FBOs and was significant only in FBO 1, trial 1 (Fig. 1**b**). Conversely, synbiotic administration in feed (group F) appeared to have an inverse (negative) effect on average broiler weight, although the differences were not statistically significant (Fig. 1**b**).

**Table 1:** Broiler production parameters.

Study group	Trial	Barn	Flock size (number of broilers per barn)	Average age at slaughter [days]	Total animal loss [%]	Average body weight [kg]	Feed conversion rate	European efficiency factor
	**FBO 1**							
**CTRL**	1	1	19,400	39.5	2.22	2.15	1.70	315
		2	21,350	38.3	2.20	2.19	1.75	321
	2	1	19,500	39.1	4.70	2.31	1.66	339
		2	18,450	38.9	3.97	2.34	1.66	349
**W**	1	1	19,450	38.4	3.72	2.26	1.70	333
		2	20,100	38.5	4.31	2.28	1.66	342
	2	1	19,600	38.1	2.91	2.32	1.58	374
		2	19,100	39.3	3.63	2.40	1.66	356
**F**	1	1	20,500	39.0	1.94	2.21	1.75	318
		2	20,600	39.0	1.74	2.24	1.79	317
	2	1	19,300	36.9	4.31	2.17	1.64	344
		2	20,200	39.3	2.76	2.20	1.76	310
	**FBO 2**							
**CTRL**	1	1	12,500	38.0	2.30	2.16	1.68	330
		2	12,500	36.0	2.52	2.00	1.65	328
	2	1	12,000	38.0	3.63	2.25	1.73	330
		2	11,000	40.0	3.06	2.39	1.65	351
**W**	1	1	12,500	38.0	2.26	2.17	1.68	332
		2	12,500	38.0	2.22	2.17	1.69	330
	2	1	12,000	38.0	3.83	2.19	1.68	330
		2	11,000	39.0	5.05	2.31	1.70	331
**F**	1	1	12,500	36.0	2.58	1.64	1.64	320
		2	12,500	38.0	1.87	1.69	1.69	322
	2	1	12,000	38.0	4.68	2.14	1.62	331
		2	11,000	40.0	1.91	2.25	1.69	326

Note: **W, F** and **CTRL** denote the synbiotic supplementation in water, feed or no supplementation (control group), respectively. Two trials were performed by each food business operator (**FBOs 1** and **2**).

### *Effect of synbiotic administration on gut colonization with Campylobacter jejuni and Salmonella* Infantis

All samples that tested positive for *Salmonella* spp. by real-time PCR were also cultured and serotyped according to ISO 6579-1:2017 and ISO 6579-3:2014 and confirmed as *S.* Infantis (data not shown). In some cases, *S.* Infantis was present in 3-day-old broilers (AG 1), but most frequently at the age of 19–22 days (AG 3). *C. jejuni* most frequently colonized broilers after 19–22 days of age. Surprisingly, *C. jejuni* was undetected in all barns of FBO 2 in trial 2.

We observed a very limited effect of synbiotic administration on the gut colonization with *C. jejuni* or *S.* Infantis (Table 2). Synbiotic administration in feed (group F) significantly reduced the gut colonization with *S.* Infantis from 19.5 to 11.3% (Fisher’s exact test, *p* = 0.045), whereas no such effect was observed in group W (Fig. S1). In addition, no significant effect of synbiotic administration on *C. jejuni* colonization was observed (Fig. S1).

**Table 2:** Real-time PCR-based detection of *Campylobacter jejuni* and *Salmonella* spp. in broiler cecal contents.

Study group	Trial	Barn	AG 1	AG 2	AG 3	AG 4
	**FBO 1**					
**CTRL**	1	1	*neg*	*neg*	SAL	SAL, CJ
		2	*neg*	*neg*	SAL	SAL, CJ
	2	1	*neg*	*neg*	SAL	SAL, CJ
		2	*neg*	*neg*	SAL	SAL, CJ
**W**	1	1	*neg*	*neg*	SAL	SAL, CJ
		2	*neg*	*neg*	SAL	SAL, CJ
	2	1	*neg*	*neg*	SAL	SAL, CJ
		2	*neg*	*neg*	SAL	CJ
**F**	1	1	*neg*	*neg*	SAL	SAL, CJ
		2	*neg*	*neg*	*neg*	SAL, CJ
	2	1	*neg*	*neg*	SAL	SAL, CJ
		2	*neg*	*neg*	*neg*	SAL, CJ
	**FBO 2**					
**CTRL**	1	1	*neg*	*neg*	SAL, CJ	SAL
		2	*neg*	*neg*	SAL, CJ	*neg*
	2	1	SAL	SAL	*neg*	SAL
		2	*neg*	SAL	SAL	*neg*
**W**	1	1	*neg*	*neg*	SAL, CJ	SAL, CJ
		2	*neg*	*neg*	SAL, CJ	SAL
	2	1	*neg*	*neg*	SAL	*neg*
		2	SAL	SAL	*neg*	SAL
**F**	1	1	*neg*	*neg*	SAL	SAL, CJ
		2	*neg*	*neg*	*neg*	CJ
	2	1	SAL	*neg*	*neg*	*neg*
		2	*neg*	SAL	SAL	SAL

Note: **W, F** and **CTRL** denote synbiotic administration in water, feed or no supplementation (control group), respectively. Detection of *Campylobacter jejuni* (CJ) or *Salmonella* spp. (SAL) in cecal contents was performed using real-time PCR. Two trials were carried out at each food business operator (**FBOs 1** and **2**). Broilers were sampled at 3 (AG 1), 7–8 (AG 2), 19–22 (AG 3) and 32–36 (AG 4) days of age. AG, age group. All samples that tested positive by *Salmonella* spp. real-time PCR were subsequently cultured, serotyped and confirmed as *S*. Infantis (data not shown).

### Effect of the synbiotic on broiler gut microbiota

**Fig. 2:**
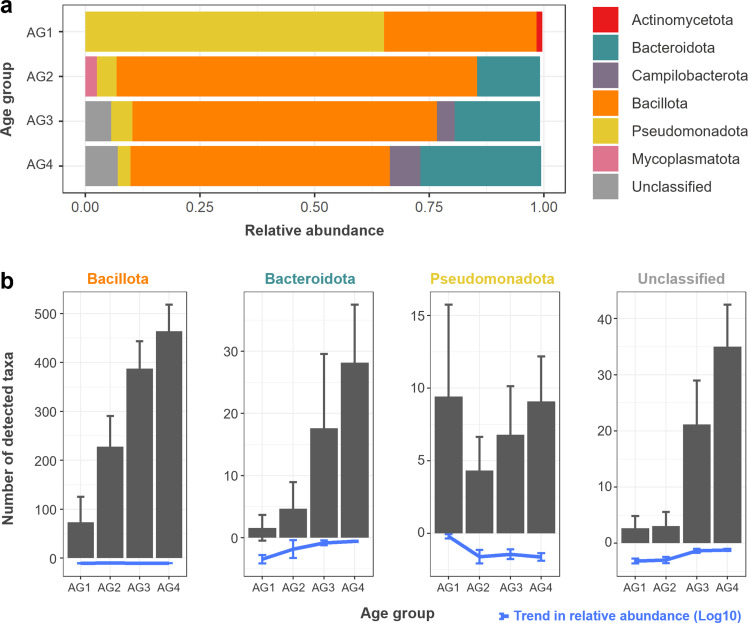
**Cecal microbiota composition**. Relative abundance of bacterial phyla during different stages of the broiler production cycle **(a).** Relative abundance of bacterial phyla in four age groups (AGs 1–4) **(b).** Community richness based on the observed ZOTUs (mean + SD) in different AGs for the three major phyla and unclassified ZOTUs. The blue line at the bottom represents the dynamics in relative abundance (log_10_[mean ± SD]). Abbreviations: ZOTU, zero radius operational taxonomic unit; SD, standard deviation.

We performed 16S rRNA amplicon sequencing to investigate the effect of the synbiotic PoultryStar on broiler cecal microbiota. The changes in bacterial microbiota composition during the broiler production cycle were most pronounced in the first half of the production cycle and stabilized in the second half of the production cycle (Fig. 2**a**). After hatching (AG 1), *Pseudomonadota* were the predominant phylum, with *Escherichia* and *Klebsiella* being the predominant genera. In the later stages of the production cycle, *Pseudomonadota* decreased and were replaced by *Bacillota* and *Bacteroidota* in adult broilers. *Bacillota* was the most diverse phylum throughout broiler development, with a total of 1987 ZOTUs detected, and approximately 450 ZOTUs per broiler observed at the final production cycle (AG 4, Fig. 2**b**). Although *Bacteroidota* was the second most abundant phylum overall, its diversity was notably lower (approximately 30 ZOTUs per broiler) than that of *Bacillota* (Fig. 2**b**). These findings were consistent across both study replicates on both FBOs (Fig. S2). Interestingly, at the later stages of the production cycle (AGs 3 and 4), an increase in representatives unclassified at the phylum level was observed, suggesting the presence of potentially important but poorly characterized bacterial taxa (Fig. 2**b**).

PERMANOVA was used to assess the contribution of different variables to the overall variation in bacterial community composition. The results showed that FBO, trial, AG, *C. jejuni* colonization status (determined as present or absent by real-time PCR) were all significantly associated with microbiota composition (*p* < 0.001; Table S1). The largest effects were attributed to AG (16.4%) and FBO (10.2%), which together explained 26.6% of the overall variation in bacterial community composition. Synbiotic administration had a smaller but still significant effect on community composition (*p* = 0.005). Conversely, broiler sex and *S.* Infantis colonization (determined as present or absent by real-time PCR) showed no association with cecal microbiota composition. Significant observations are discussed in more detail in continuation.

**Fig. 3:**
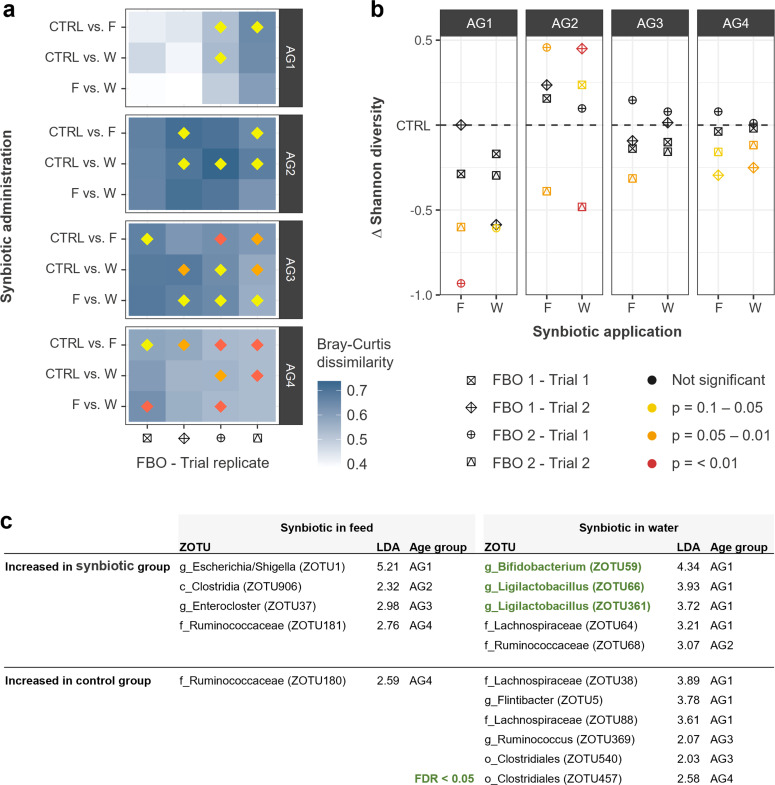
**Effect of synbiotic administration route on microbiota. (a)** Beta diversity analysis using Bray-Curtis dissimilarities comparing study groups in different age groups (AGs), separately for each FBO and trial. Color in the heat diagram represents the mean Bray-Curtis dissimilarity, whereas the diamonds inside indicate the statistical significance obtained with ANOSIM. Color coding of significance is described in the legend. **(b)** Difference in alpha diversity between the control group (CTRL) and the groups that were administered the synbiotic either in feed (F) or water (W). Alpha diversity is presented as Shannon diversity index. Four trials are labeled with different shapes to highlight the differences among them. Statistical significance is color-coded as described in the legend. **(c)** The population-level analysis (LEfSe) lists differentially abundant ZOTUs when comparing groups F and W with the control group (CTRL) separately for each AG (column Age). ZOTUs with a false discovery rate (FDR) of < 0.01 are highlighted in green. Abbreviations: FBO, food business operator; ZOTU, zero radius operational taxonomic unit; LDA, linear discriminant analysis; ANOSIM, analysis of similarities; LEfSe, Linear discriminant analysis Effect Size.

***Effect of synbiotic administration route.*** Synbiotic administration had a significant effect on cecal microbiota composition, and the microbiota differed both between the administration routes (group W vs. group F) and between trials (trial 1 vs. trial 2) (Fig. 3**a**). After the high dispersal observed in AG 2 (greater Bray-Curtis dissimilarities marked by darker shades of blue, Fig. 3**a**), microbiota stabilized in AGs 3 and 4 and showed stronger significant differences between the study groups (denoted with different colored diamonds based on ANOSIM significance, Fig. 3**a**). This was also confirmed by AMOVA analysis, where the number of significant differences between the study groups increased with broiler age (Table S2). Synbiotic administration had the most pronounced effect in FBO 2, with the strongest significance observed in later stages (AGs 3 and 4). The strongest effect was observed in study group F, which differed significantly from the control group at the final stage (AG 4) in all four trials (Fig. 3**a**).

Alpha diversity analysis demonstrated the disturbance introduced by synbiotic application (Fig. 3**b**). The synbiotic caused a ‘yo-yo’ effect, with an initial decrease in diversity in synbiotic treatments compared with the control group, followed by a compensatory increase in AG 2 and stabilization in AGs 3 and 4. Notably, alpha diversity in AG 1 was significantly inversely correlated with *E. coli* abundance (Pearson’s *r* = –0.84, *p* < 0.001; Fig. S3), though no phenotypical characteristics were associated with this observation. Finally, diversity stabilized near the values observed in control group but showed a slight decrease in both synbiotic experimental groups in AG 4 in two out of four trials. In FBO 2, trial 2, alpha diversity was significantly lower than in the other AG 2 replicates (Fig. 3**b**). Collectively, the results of alpha diversity analysis suggest that the effect of the synbiotic is trial-dependent.

The LEfSe analysis revealed that the differentially abundant taxa distinguishing groups F and W varied substantially across study replicates. Fig. 3**c** shows the ZOTUs for which the synbiotic-associated differential abundance was consistent across all four trials. Of the listed ZOTUs, only three were significant after correcting for false discovery rate (FDR < 0.05), corresponding to the ZOTUs obtained from sequencing of the synbiotic PoultryStar Sol (*Bifidobacterium* [ZOTU59] and *Ligilactobacillus* [ZOTU66 and ZOTU361]). Since 16S rRNA amplicon sequencing lacks strain-level resolution, probiotic strains could not be reliably distinguished from the native microbiota. Only the *Bifidobacterium* ZOTU59 could be unequivocally linked to the synbiotic, as it was absent in the control group in all but one trial (Fig. S4). Abundance of these taxa increased in AG 1, but only in group W. This may be related to the administration route used since broilers in group W were also sprayed with a synbiotic-supplemented water solution at housing, potentially leading to contamination at the time of sample collection.

***Bacterial taxa associated with Campylobacter jejuni colonization.*** Regardless of the administration route, *C. jejuni* gut colonization was significantly associated with microbiota composition (PERMANOVA, explaining 1.2% of the overall variation, *p* < 0.001). The bacterial taxa associated with pathogen colonization differed between trials, particularly between the two FBOs. Nevertheless, *C. jejuni* colonization was negatively correlated with the presence of *Lactobacillus* (ZOTU27) and an unclassified bacterium from the phylum *Bacteroidota* (ZOTU174) in different trials in both FBOs (Fig. S5). The correlation was reflected in a significant decrease in the relative abundance of these two bacterial taxa in broilers colonized with *C. jejuni*. In addition, the relative abundance of *Lactobacillus* (ZOTU27) was directly negatively correlated with *C. jejuni* load, as determined by real-time PCR (Pearson's *r* = –0.30, *p* = 0.023, Fig. S5).

***Bacterial taxa associated with broiler weight at different age groups.*** Several bacterial taxa were correlated with broiler weight, and these correlations varied across AGs. In the early and middle stages of the production cycle, predominantly negative correlations were observed, especially with genera such as *Escherichia* and *Butyricimonas*. In contrast, predominantly positive correlations were observed in AG 4, including various representatives from the families *Bacteroidaceae* and *Lachnospiraceae* as well as the genus *Ligilactobacillus* (Fig. S6). At the phylum level, *Bacteroidota*-to-*Bacillota* ratio showed a production cycle-dependent effect on broiler weight. In AG2, broiler weight was positively correlated with increased *Bacteroidota* (*p* < 0.001), whereas in AG 3, it was negatively correlated with *Bacteroidota*-to-*Bacillota* ratio (*p* < 0.001). In AG 4, the *Bacteroidota*-to-*Bacillota* ratio showed no correlation with broiler weight. The correlation between bacterial community diversity and weight gain was non-significant.

## Discussion

Poultry represents a key reservoir for *Campylobacter* and *Salmonella*, two leading foodborne pathogens. Broiler carcasses are often contaminated during slaughter, with leakage of intestinal content during evisceration being an important source of contamination (Chlebicz and Śliżewska, 2018; EFSA and ECDC, 2023). Control measures to prevent pathogen introduction include farm biosecurity, vaccination, probiotics and slaughterhouse hygiene (Zamojska et al., 2021). In this study, *S.* Infantis was detected early in the production cycle (three days; AG 1), whereas *C. jejuni* appeared later (19–22 days; AG 3), consistent with previous studies (Al Hakeem et al., 2022; Soffritti et al., 2025). *C. jejuni* was not detected in any barns of FBO 2 in trial 2, including the control group, suggesting that its absence was coincidental rather than an effect of synbiotic administration. This observation was unusual, since the proportion of *C. jejuni*-positive broiler flocks in Slovenia can be as high as 70% (AFSVSPP, 2025) and the barns of FBO 2 had tested positive in several previous production cycles, including in trial 1.

The present field study, which deals with the effect of the synbiotic PoultryStar on broiler production performance, *C. jejuni* and *S.* Infantis colonization and composition of cecal microbiota was performed on two FBOs under intensive farming conditions during the entire production cycle. Previous studies have relied on controlled challenge tests with intentionally infected animals, often involving a limited number of animals or shortened experimental periods that did not cover the entire production cycle (Ghareeb et al., 2012; Guyard-Nicodeme et al., 2016; Luoma et al., 2017; Shanmugasundaram et al., 2019; Mortada et al., 2020; Sobotik et al., 2021; Cason et al., 2023; Drauch et al., 2025). Only a few field studies have examined the impact of probiotics on pathogens in broilers, and none have investigated the synbiotic PoultryStar (Smialek et al., 2018; Soffritti et al., 2025). The choice of the synbiotic/probiotic preparation can also significantly affect study outcomes, limiting comparability between studies (Mekonnen et al., 2024). In this study, the synbiotic PoultryStar, marketed to improve gut health and performance, had a limited impact on reducing *C. jejuni* and *S.* Infantis colonization. Feed-only administration reduced the gut colonization with *S.* Infantis from 19.5 to 11.3%, but no effect was observed regarding *C. jejuni* colonization. Several studies have reported that the synbiotic PoultryStar reduces *C. jejuni* colonization (Ghareeb et al., 2012; Guyard-Nicodeme et al., 2016; Cason et al., 2023), whereas others have found no effect on *Campylobacter coli* colonization (Mortada et al., 2020). Previous *in vivo* studies on *Salmonella* colonization in the gut of broilers and laying hens showed a decrease in *Salmonella* Enteritidis and *Salmonella* sp. load (Luoma et al., 2017; Shanmugasundaram et al., 2019; Sobotik et al., 2021). A recent challenge test investigating the effect of the synbiotic PoultryStar on *S.* Infantis colonization and shedding showed a reduction in *S.* Infantis loads in the cecum, ileum and spleen, but no influence on pathogen shedding (Drauch et al., 2025). *S.* Infantis carrying the pESI megaplasmid encodes genes that increase host fitness and environmental persistence (Papić et al., 2022), which may explain the observed differences in responses among different *Salmonella* serovars. It should also be noted that different variations of the PoultryStar synbiotic exist. The preparation used in this study comprised three different strains, while other studies have used preparations with four to five strains (Ghareeb et al., 2012; Guyard-Nicodeme et al., 2016; Cason et al., 2023; Drauch et al., 2025). Multi-strain probiotics are thought to be more effective than single-strain formulations in enhancing probiotic efficacy, but its dose or the add-on regime has not been clearly defined yet (Halder et al., 2024).

In this study, water-only administration showed a significant increase in EEF compared with the feed-only administration, but the increase was not significantly different from that of the control group. All the other production parameters did not differ significantly between the study groups. In previous studies using the synbiotic PoultryStar, additional beneficial effects on growth parameters (e.g. body weight, feed intake and FCR) were observed (Mountzouris et al., 2010; Ghareeb et al., 2012; Mohammed et al., 2018; Sobotik et al., 2021; Prentza et al., 2023; Ma et al., 2025). However, other studies using the same synbiotic reported no effects on performance parameters (Cason et al., 2023; Boyner et al., 2025).

The present results indicate that the effect of probiotic administration on the gut microbiota is highly influenced by the administration route, as previously hypothesized (Karimi Torshizi et al., 2010). In this study, the administration route influenced outcomes: feed-only administration reduced gut colonization with *S.* Infantis, whereas water-only administration tended to improve production parameters. Similarly, a recent study (Drauch et al., 2025) compared three different administration routes of the synbiotic PoultryStar (feed, water and combined) and showed different effects on the reduction of *S.* Infantis cecal load, broiler weight and gut microbiota. Overall, combined administration was the most recommended, followed by administration in water. However, the greatest reduction in *S.* Infantis cecal load was observed in the group receiving the synbiotic in water. In addition, Ma et al. (2025) showed a beneficial effect on growth parameters when the PoultryStar synbiotic was administered in water compared with the administration in feed.

In this study, no significant effect of probiotic on *C. jejuni* colonization was observed. However, two bacterial taxa, belonging to genus *Lactobacillus* phylum *Bacteroidota*, showed negative correlation with *C. jejuni* colonization and could therefore contribute to colonization resistance against this pathogen. Several other studies have also reported that certain bacterial taxa are negatively correlated with *Campylobacter* colonization rate in broilers (Ocejo et al., 2019; Di Marcantonio et al., 2022; Pang et al., 2023a, 2023b). The known antagonistic effects of various *Lactobacillus* species against *C. jejuni* include growth inhibition (Dec et al., 2018; Catacutan et al., 2022; Wishna-Kadawarage et al., 2024), inhibition of virulence factor expression (Alizadeh et al., 2021) and reduction in adherence and invasion (Šikić Pogačar et al., 2020). Beyond direct antagonism, *Lactobacillus* species can modulate immune responses, enhancing the activity of macrophages and other immune cells against *C. jejuni* (Taha-Abdelaziz et al., 2019). On the other hand, the novel potential *C. jejuni* antagonist from the phylum *Bacteroidota* requires isolation and experimental validation, as no such studies have been reported to date.

Cecal microbiota was dominated by three phyla (*Bacillota, Bacteroidota* and *Pseudomonadota*) and showed significant shifts between different stages of the production cycle, with *Bacillota* showing the highest abundance and taxonomic richness. Several studies have reported such age-related shifts in cecal microbiota of broilers (Xiao et al., 2021; Yin et al., 2023), but other host-related factors and geographical origin of birds are also known to influence cecal microbiota. For example, Zhou et al. (2021) reported a relatively stable *Bacillota*-dominated microbiome, whereas Clavijo et al. (2022) reported a higher *Bacteroidota*-to-*Bacillota* ratio throughout development. While a previous meta-study linked *Bacillota*-dominated microbiota with increased food intake and higher final body weight in chickens (Deryabin et al., 2024), the present results suggest a production stage-dependent association between broiler weight and the *Bacteroidota*-to-*Bacillota* ratio, with no effect observed in the final stage of the production cycle. Deryabin et al. (2024) also found a positive correlation between production efficiency and microbiota diversity, a relationship that was not observed in this study.

This study supports previous findings that probiotic administration alters gut microbiota (Mikulski et al., 2012; Shehata et al., 2020; Drauch et al., 2025). Here, a positive correlation between broiler weight and several bacterial taxa belonging to orders *Bacteroidales* and *Bacillales* was observed, which is only partially consistent with previous studies reporting positive correlations with *Alistipes* (Marcolla et al., 2023) and various *Bacillota* (Deryabin et al., 2024). Such discrepancies are not unexpected, as poultry gut microbiota is shaped by different external factors, including feed composition, stocking density, poultry breed, geographical location and pathogen colonization, all of which complicate direct comparison between related studies (Guardia et al., 2011; Oakley et al., 2014; Stanley et al., 2014; Metzler-Zebeli et al., 2018; Haberecht et al., 2020; Zhou et al., 2021). Our experimental design furthermore enabled a systematic evaluation of how synbiotic preparation affected cecal microbiota, both across different farms and in consecutive experiments conducted on the same farm. Notably, even repeated trials yielded different broiler production parameters and gut microbiota alterations in response to symbiotic supplementation. To our knowledge, no prior studies have highlighted such variability in probiotic effects within a single farm, underscoring the need of long-term monitoring with multiple trial repetitions to evaluate sustained impact of probiotic on production outcomes.

In conclusion, the present results demonstrate that while the synbiotic used in this study significantly influenced certain production parameters and cecal microbiota composition in broilers, other rearing-related factors such as the administration route, production cycle, farm-specific conditions and trial repetition exerted an even stronger influence on the outcomes, particularly on microbiota. Notably, unique microbial signatures were observed in consecutive trial replicates on the same farm, underlining the importance of comprehensive, repeated and standardized evaluation of probiotic efficacy. Tailoring probiotics to the specific conditions of individual farms may offer a more effective alternative to generic commercial preparations in improving production outcomes.

## Funding

This study was financially supported by the Slovenian Research and Innovation Agency (“Use of probiotics to control contamination of poultry farms with campylobacters and salmonella” – project no. J4-1774; “Animal health, environment, and food safety” – research core funding no. P4-0092; “Gut microbiota in health and disease” – research core funding no. P3-0387).

## CRediT authorship contribution statement

**Jana Avberšek:** Writing – original draft, Project administration, Investigation, Data curation, Conceptualization. **Aleksander Mahnič:** Writing – original draft, Visualization, Investigation, Formal analysis, Data curation. **Darja Kušar:** Writing – original draft, Investigation. **Bojan Papić:** Writing – original draft, Formal analysis. **Olga Zorman Rojs:** Writing – review & editing, Resources, Project administration, Conceptualization. **Tomaž Knafelc:** Writing – review & editing, Resources, Conceptualization. **Jasna Perc:** Writing – review & editing, Supervision, Resources, Project administration. **Maja Rupnik:** Writing – review & editing, Supervision, Funding acquisition. **Matjaž Ocepek:** Writing – review & editing, Supervision, Resources, Funding acquisition, Conceptualization.

## Disclosures

The authors declare the following financial interests/personal relationships which may be considered as potential competing interests: Matjaz Ocepek reports equipment, drugs, or supplies was provided by BIOMIN Holding GmbH. If there are other authors, they declare that they have no known competing financial interests or personal relationships that could have appeared to influence the work reported in this paper.

## Appendix Supplementary materials

Image, application 1

## Footnotes

Supplementary material associated with this article can be found, in the online version, at doi:10.1016/j.psj.2025.106353.
