# Supplementary material for: Administration route and trial repetition shape the effects of a commercial synbiotic on broiler production performance, cecal microbiota and pathogen colonization
Source: Poult Sci. 2025 Dec 28;105(3):106353. doi: 10.1016/j.psj.2025.106353 (PMC12804107; doi:10.1016/j.psj.2025.106353)
Supplement: Supplementary file 1 [file mmc1.docx]

**Table S1 Effect of different variables on microbiota composition.** Permutational ANOVA analysis (PERMANOVA, 1000 iterations). Significance is denoted as: *** < 0.001, ** 0.01–0.001. FBO, food business operator.

|  | **R^2^** | ***p* value** |  |
| --- | --- | --- | --- |
| **FBO (FBO 1, FBO 2)** | 0.10174 | 0.000999 | *** |
| **Trial repetition (Trial 1, Trial 2)** | 0.02152 | 0.000999 | *** |
| **Age group (AG 1, AG 2, AG 3, AG 4)** | 0.16391 | 0.000999 | *** |
| **Synbiotic (Control, Feed, Water)** | 0.00809 | 0.004995 | ** |
| **Sex (Male, Female)** | 0.00239 | 0.360639 |  |
| ***Salmonella* Infantis colonization (Yes, No)** | 0.00285 | 0.121878 |  |
| ***Campylobacter jejuni* colonization (Yes, No)** | 0.01155 | 0.000999 | *** |

**Table S2 Analysis of molecular variance (AMOVA) comparing the study groups according to the synbiotic administration route.** The results are shown for each combination of age group (AG) and trial repetition. The numbers in the table denote the *p*-values. FBO, food business operator.

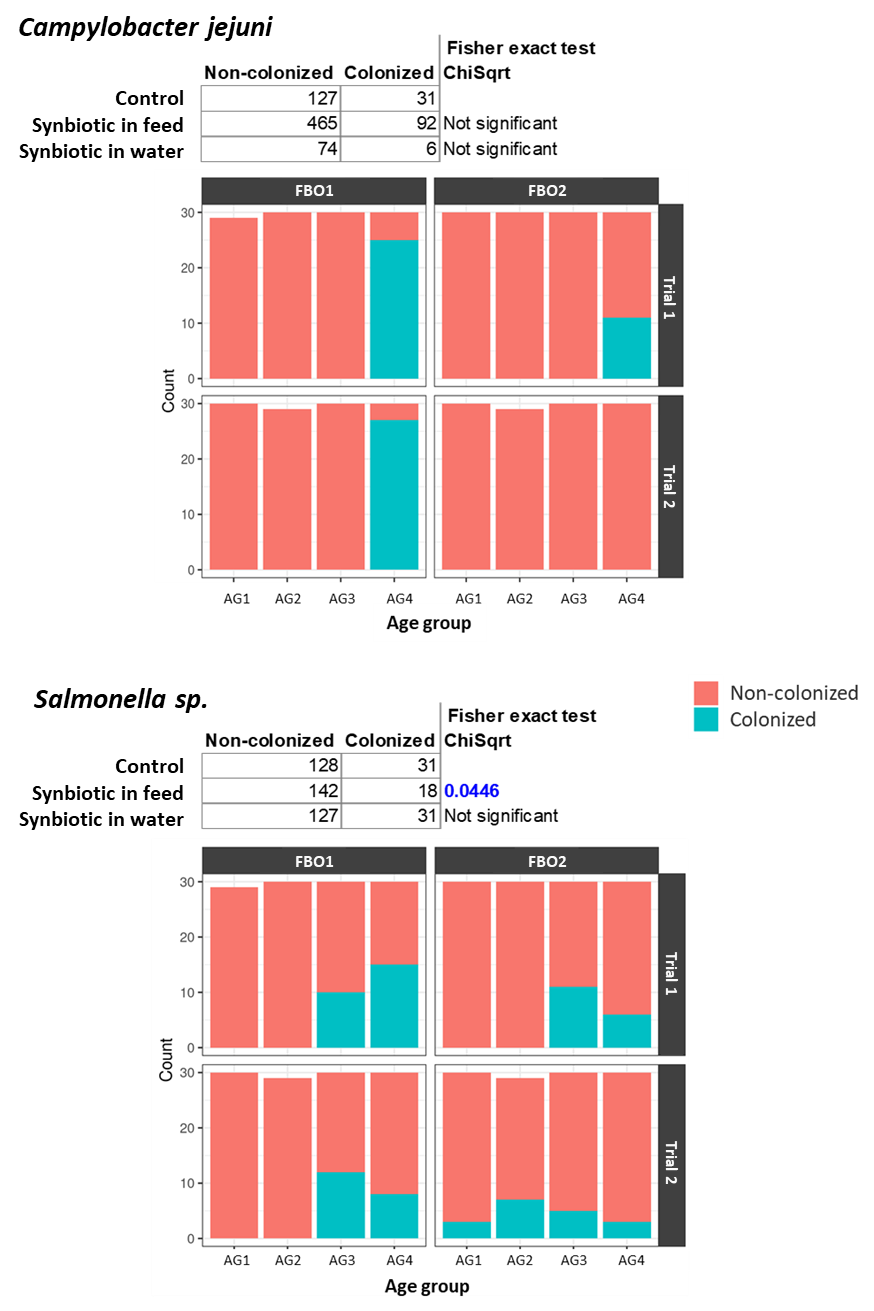


**Fig. S1. *Salmonella* Infantis and *Campylobacter jejuni* gut colonization.** Fisher's exact test was used to evaluate the proportion of colonized broilers for each food business operator (FBOs 1 and 2) and synbiotic administration route (water vs. feed). The tables above the plots show the number of colonized and non-colonized broilers, whereas the stacked barplots represent the number of broilers, colored by *C. jejuni* (top) and *S.* Infantis (bottom) colonization status.


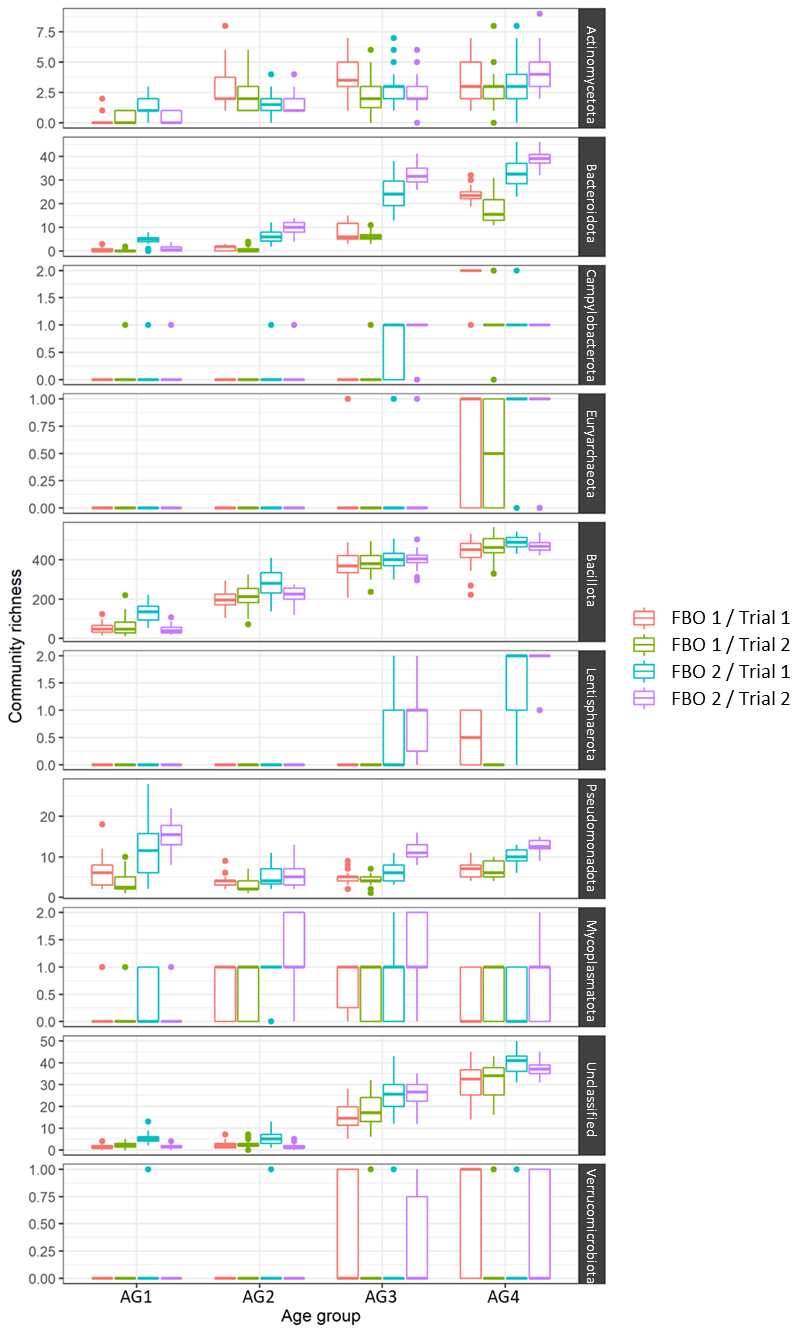


**Fig. S2. Microbial richness (expressed as the number of observed ZOTUs) for each detected bacterial phylum.** Box plots are color-coded according to the food business operator (FBO) and trial repetition.


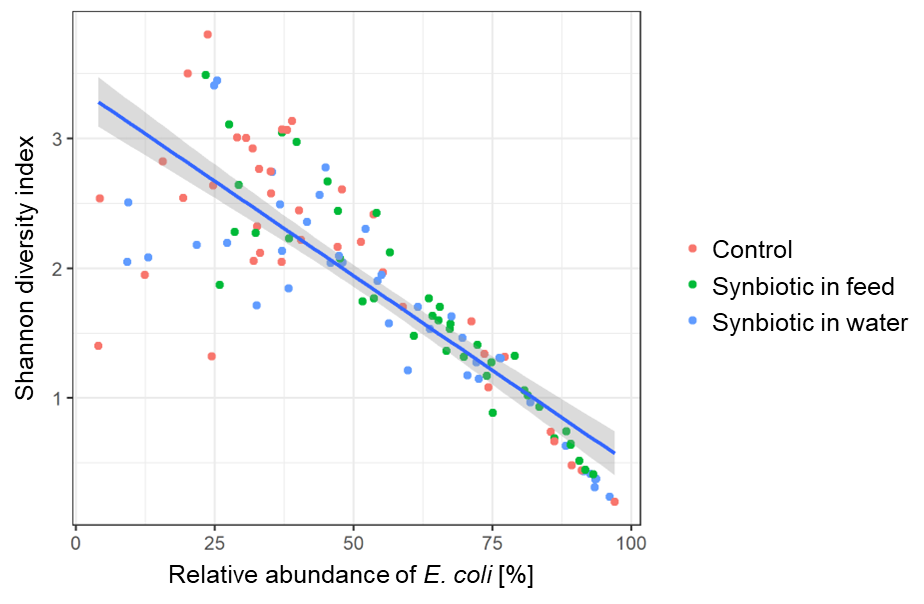


**Fig. S3.** Correlation between the Shannon diversity index and the relative abundance of *Escherichia coli* in broilers of age group 1 (AG 1). The color coding shows the different synbiotic administration routes. The blue line shows the linear regression, with the 95% confidence interval shaded grey.


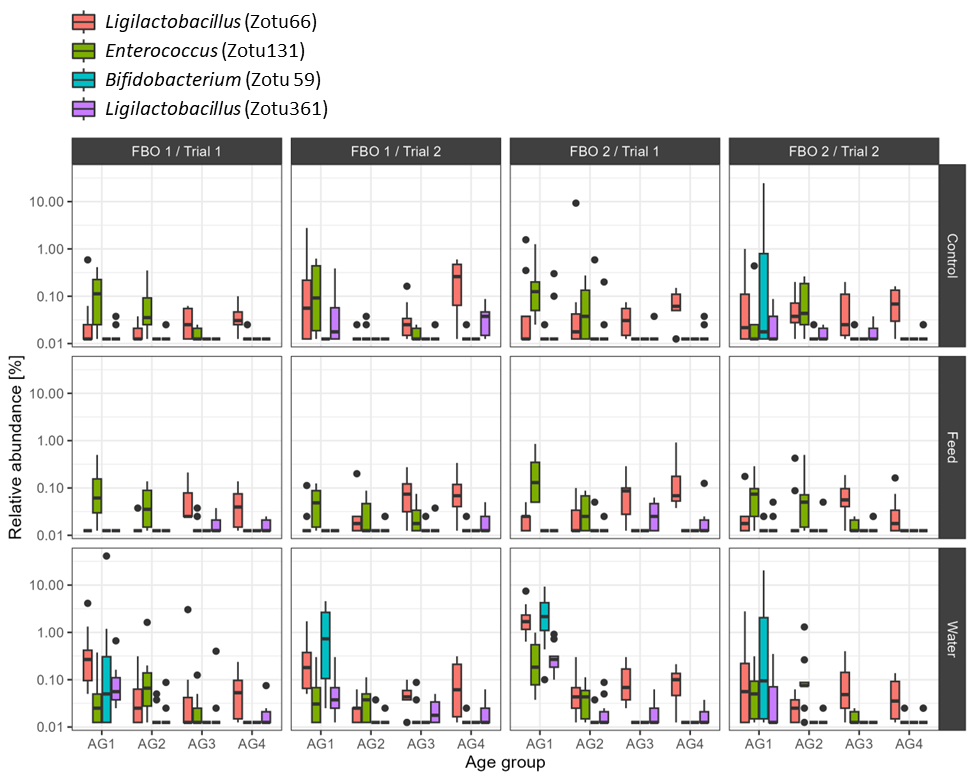


**Fig. S4.** Dynamics of PoultryStar Sol synbiotic-associated taxa in broiler cecal microbiota. Relative abundances of four synbiotic-associated ZOTUs are shown for each trial repetition and administration route across the different broiler age groups (AGs).


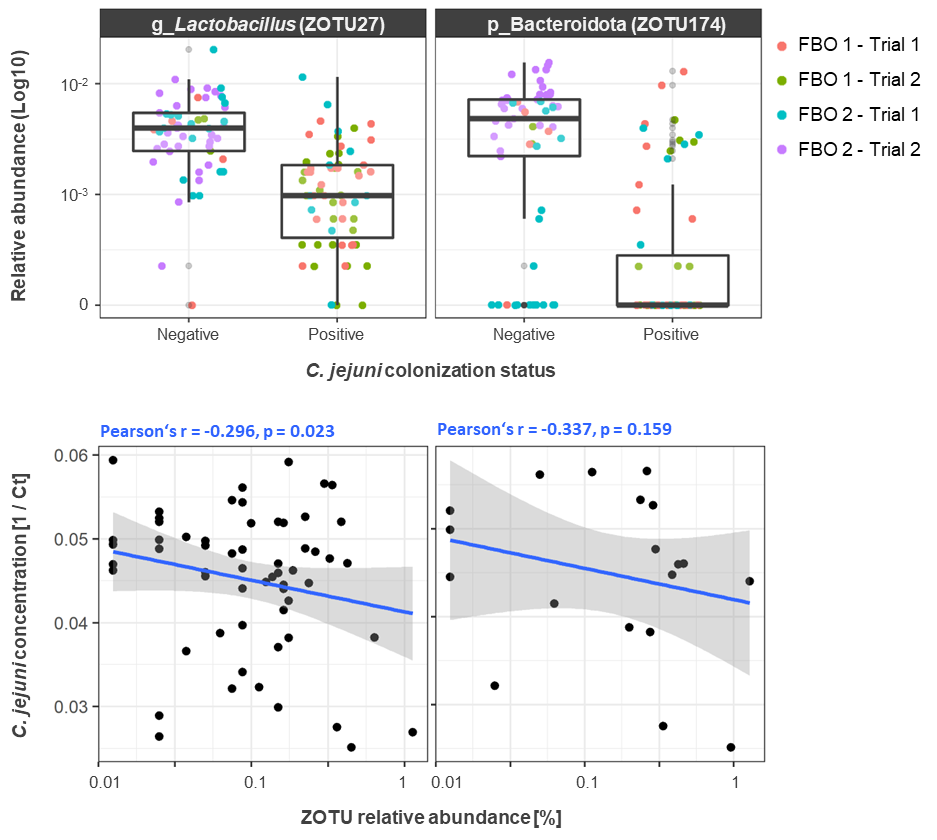


**Fig. S5.** Relative abundances of the two ZOTUs negatively correlated with gut colonization with *Campylobacter jejuni*. The box plots (upper figures) show the cumulative abundance of the two ZOTUs, whereas the coloring of the dots represents the trial repetition and the food business operator (FBO). The dot plots (lower figures) represent the correlation between the *C. jejuni* load determined by the species-specific real-time PCR and the respective ZOTU. Only positive values for both taxa (i.e. samples in which both ZOTUs were present) were used to assess the direct antagonistic relationship.

**
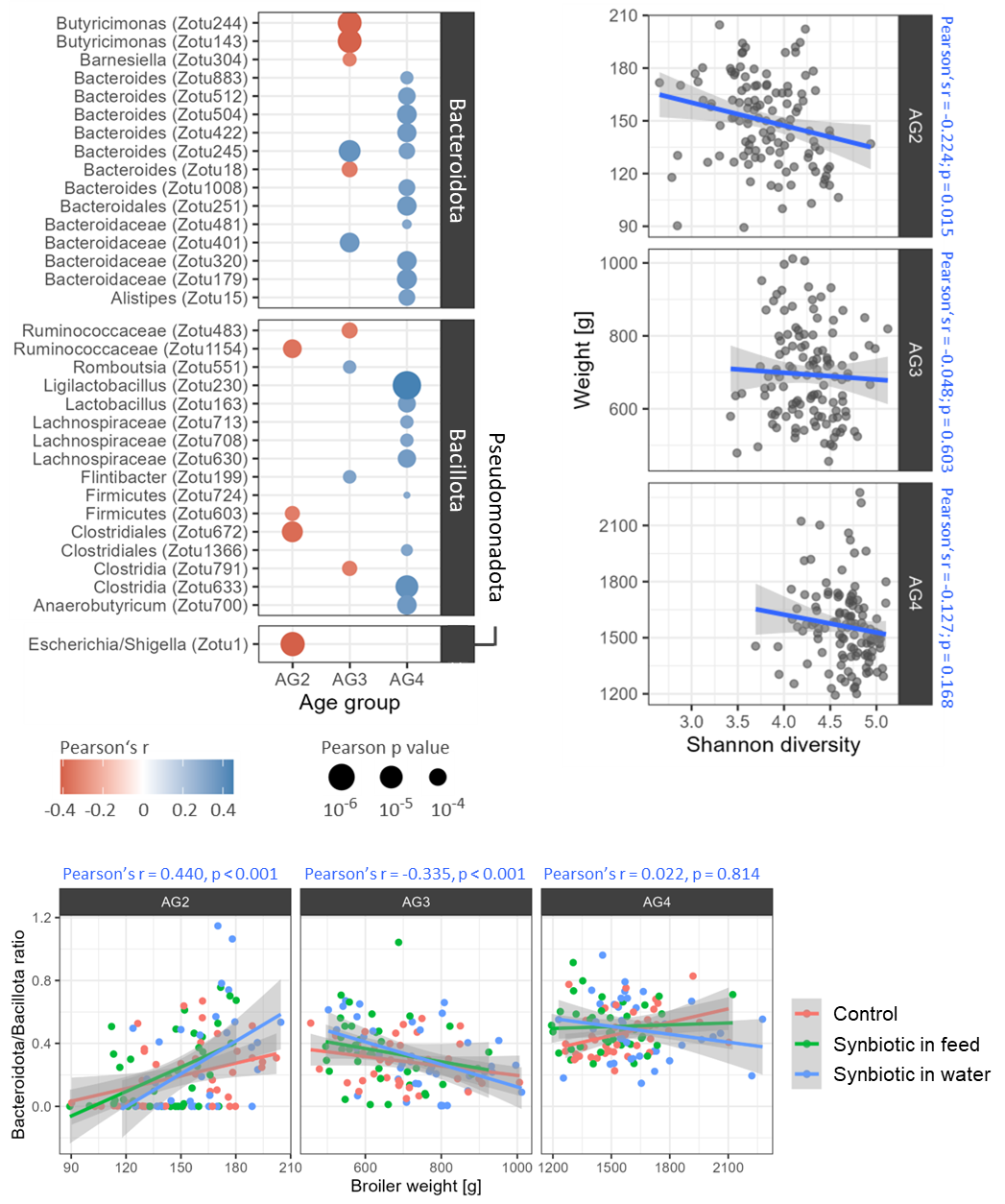
**

**Fig. S6.** Correlation between microbiota composition and broiler weight. The Pearson correlation coefficient (*r*) between broiler weight and ZOTU relative abundance (false discovery rate < 0.05) is shown separately for each age group (AG). The color scale indicates Pearson's *r*, whereas the size of the dots indicates significance (left). The correlation between broiler weight and the Shannon diversity index is shown separately for each AG (right). The Pearson's test values are shown to the right of each chart. The *Bacteroidota*-to-*Bacillota* ratio is shown separately for each AG and is color-coded according to the synbiotic administration route (bottom).
